# Antioxidant Protection of Paper Heritage Objects with 4-Amino-2,2,6,6-Tetramethylpiperidine

**DOI:** 10.3390/ma16093351

**Published:** 2023-04-25

**Authors:** Katarína Vizárová, Soňa Malečková, Radko Tiňo, Milena Reháková, František Kačík

**Affiliations:** 1 Faculty of Food and Science Technology, Slovak University of Technology, Radlinského 9, 812 37 Bratislava, Slovakia; 2 Faculty of Wood Sciences and Technology, Technical University in Zvolen, T. G. Masaryka 24, 960 53 Zvolen, Slovakia

**Keywords:** acid paper, stabilization, piperidine, magnesium, hexamethyldisiloxane

## Abstract

In protecting cultural heritage, deacidification is used to stabilize acidic papers, ensuring the neutralization of acids present in the paper. But in the process of aging, several mechanisms of degradation occur simultaneously. Therefore, substances used to stabilize the paper should contain an antioxidant component in addition to the deacidifying component. The effect of the antioxidant (hindered amine light stabilizers—HALS type) on the stabilization of lignin-containing acid papers during accelerated aging was studied in this work. The effective deacidification component was magnesium; the solvent was hexamethyldisiloxane (HMDSO). The 4-amino-2,2,6,6-tetramethylpiperidine series of hindered amine light stabilizers (HALS) was selected as appropriate for creating a modifying system based on HMDSO. The modification system was tested on samples of a model test acid lignin-containing paper (NOVO) and naturally aged acidic paper from the real book. The addition of substances to the proposed deacidification system has a favorable effect on stabilizing the pH during aging and provides the inhibition of the oxidative degradation products and stabilization of the cellulose degree of polymerization. It was confirmed that the application of the system containing deacidification and an antioxidant component may increase the effectiveness of the protection of acid groundwood paper heritage objects. Adding a tested antioxidant to the proposed deacidification system has a favorable effect on stabilizing the pH value for a long time in accelerated aging.

## 1. Introduction

One of the most important factors to consider in permanent materials degradation is oxidation, induced by light irradiation, the presence of atmospheric oxygen, pollutants, radicals, transition metal ions, and other oxidizing agents. In the context of the study of degradation mechanisms and conservation options, significant attention is devoted to paper as a material of cultural heritage and an information carrier. Paper is a heterogeneous sheet material characterized by a fibrous structure. The basic component of paper fibers is cellulose, which undergoes degradation during aging due to several mechanisms. Upon oxidative degradation, various products, such as carbonyls, carboxyls, and acids, are formed. Acidic products are a source of intense degradation by acid hydrolysis [1,2].

The current stabilization strategy aims to neutralize acids originating from technology and formed in material degradation during its lifetime and to create alkaline reserves to neutralize future acid products (deacidification) [3,4,5]. From the above, it is important to focus the research on the elimination of the causes of degradation, stabilization of lignocellulosic materials, and inhibition of oxidation by a suitable antioxidant. 

In practice, the protection against the oxidative degradation of paper is achieved through the use of BHT [6] or calcium phytate acting as a protector of cellulose against degradation catalyzed by iron ions [7]. The effect of halogenides [8,9] and phenols [10] was also experimentally verified and studied.

In this paper, we present a different approach: the introduction of multifunctional modification systems (MMS) [11,12,13], which allows us to solve the problem of documents, books, and other paper objects aging in a comprehensive mode, concerning ongoing degradation mechanisms (oxidation, acid hydrolysis) and consequences of degradation. In our previous work [10], we have dealt with the research and development of new stabilization systems of paper objects based on the latest knowledge from the world. To fulfil the objective mentioned above, some systems were designed and experimentally tested [14,15].

**Figure 1:**
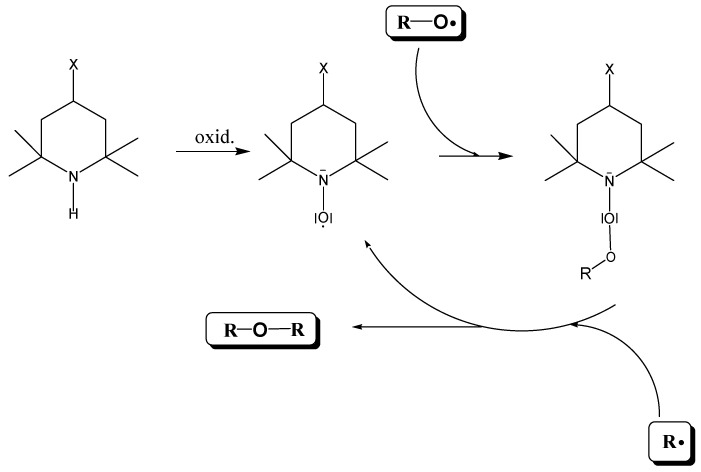
Scheme of the mechanism of conversion R· and RO· using the nitroxyl radical to non-radical products—ethers.

The selection of a suitable antioxidant and the selected concentrations were the subjects of prior research, carried out at Slovak University of Technology in Bratislava (STUBA) as part of the KnihaSK project [10]. They tested various types of structurally different compounds with various antioxidant properties [10,16], which interfere with different parts of the oxidative cyclic chain mechanism and thus retard the oxidative degradation of useful materials. The selected antioxidant that is the subject of this study is based on piperidine and comes from the group of HALS (hindered amine light stabilizers), which are converted into nitroxyl radicals. They react very readily with alkoxy radicals and give non-radical products. The significance of the given antioxidants is that neutral adducts are formed with alkoxy radicals. They can react with radicals, forming a stable ether molecule R-O-R. The beginning radical for the re-attack of another radical is restored (Figure 1) [17,18,19,20].

The advantage of HALS is the cyclic “regenerability” of the active nitroxyl radical and the fact that HALS also interferes with other parts of the oxidation of materials. In addition, there is a conversion of radical particles into non-radical products. They are photo stabilizers, quenchers, and a singlet oxygen, which greatly interfere with the auto-oxidation cycle by reducing the concentration of R· and ROO· radicals in the oxidation system [19,20,21,22]. A final advantage of the use of HALS as anti-degradants in lignocellulosic materials is the presence of lignin. Lignin as a natural antioxidant can be included in the class of donors–phenolic antioxidants. It is known from the practice of protection of polymeric materials that phenolic antioxidants are very well tolerated with HALS [18].

## 2. Materials and Methods 

As an example, we present the development of a multifunctional modification system (MMS) in a nonpolar medium. Based on the evaluation of the deacidification process and as the best available technology (BAT) [23] for the preservation of books and documents sealed in archival boxes, the Papersave process was selected [23,24,25].

### 2.1. Samples

The *NOVO model paper* had the following qualities: CTMP pulp (KLUG-CONSERVATION) with added Al_2_(SO_4_)_3_; surface pH 4.5; grammage 90 g·m^−2^. Composition: 60% CTMP pulp, 25% bleached kraft pulp, 12–15% clay, 17% lignin content.

*The real BOOK* had the following qualities: inner book, issued in 1953 (which underwent natural aging in a library deposit for 57 years); surface pH = 4.7; grammage 70 g·m^−2^; Composition: groundwood pulp and sulfite pulp in the mass ratio of 1:1, Klason lignin of 3.2%.

### 2.2. Modification Systems

An amount of 3 types of modification were used. 1. DAO3 had the following qualities: 4-amino-2,2,6,6-tetramethylpiperidine (Sigma Aldrich, St. Louis, MO, USA, 98%), in hexamethyldisiloxane (HMDSO) (Sigma Aldrich, ≥98%) with 2 concentrations (lower concentration = 0.7% wt. DAO I., higher concentration = 1% wt. DAO3 II.). 2. METE had the following qualities: magnesium ethoxide and titanium ethoxide dissolved in HMDSO (Nitrochemie Wimmis AG, Wimmis, Switzerland) (10% wt. METE in HMDSO). 3. DAO3 II. + METE had the following qualities: higher concentration of DAO3 with METE dissolved in HMDSO (a solution of DAO3 II. in HMDSO (1%) mixed with METE in HMDSO (10%) in a volume ratio of 1:1). Reference samples were unmodified NOVO model paper and the real aged BOOK.

### 2.3. Modification Technique 

The samples were modified using the patented process (patent SK287845) [26]. The samples were modified with a solution consisting of METE dissolved in HMDSO or DAO3 in HMDSO or a solution consisting of DAO3 + METE in HMDSO. After the solution was drained, the material was vacuum-dried to remove the solvent. Consequently, the sample was exposed to humidity. Samples were conditioned according to ISO 187 at (23 ± 1) °C and (50 ± 2) %RH. The samples were subsequently aged. 

### 2.4. Accelerated Aging 

All samples were subjected to accelerated aging according to standard ASTM D 6819-02 (in a circulation dryer at (98 ± 2) °C. The aging time was 0, 3, 5, 10, or 15 days. After aging, the samples were conditioned according to standard ISO 187 at (23 ± 1) °C and (50 ± 1) %RH.

### 2.5. Folding Endurance

Measurement of double folds number was performed with an instrument MIT (Tinus Olsen Testing Machine Co., Willow Grove, PA, USA, No.: 113295-5) according to standard STN ISO 5626 1999. The measurements were carried out at a load of 0.3 kg. Each sample was measured ten times. Table 1 and Table 2 show these measurements’ average values and standard deviation.

### 2.6. pH Measurement

Surface pH was measured with a digital pH meter (5310 Jenway) equipped with an electrode for surface pH measurement according to standard Tappi 529. Each sample was measured six times on different spots on the paper surface, and the average values with standard deviation were used in the text of Section 3.2. 

### 2.7. Gel Permeation Chromatography

The GPC of the tricarbanilated samples was carried out on the PLgel MIXED B column (Polymer Laboratories, Church Stratton, UK). The carbanilation procedure was carried out as previously described [27]. Tetrahydrofuran (THF) was used as an eluent, and the data were acquired with a diode array detector at 240 nm. A universal calibration was used to determine molecular weights with the constants K = 2.01 × 10^−3^, α = 0.92.

### 2.8. Fourier Transform Infrared Spectroscopy (FTIR)

The ATR-FTIR spectra were acquired using the FTIR spectrophotometer Varian Excalibur Digilab FTS 3000MX (Palo Alto, CA, USA) equipped with the ATR (Attenuated Total Reflection) adapter with single reflection diamond crystal. The measurement range was from 4000 to 600 cm^−1^; the sensitivity was 4 cm^−1^; and there were 30 scans. The background was air. The resulting measured spectrum was obtained as an average spectrum of 10 independent measurements.

## 3. Results

This work deals with the significance of multifunctional modification systems to the enhanced stability of traditional carriers of information on acid paper: books and archival documents. Four types of modification systems (METE, DAO3 I., DAO3 II., DAO3 II. + METE) were tested on model acid lignin-containing paper (NOVO) and a real book from the collections of memory funds (BOOK). The METE modification system is generally effective. It is a process of mass deacidification of Papersave [23]. The DAO3 I. and DAO3 II. modification systems represent an antioxidant in two different concentrations where the effect on oxidative activity was monitored. The DAO3 II. + METE modification system represents a developed multifunctional system whit both antioxidant and deacidification components.

### 3.1. Folding Endurance

The modification has a significant effect on reducing the brittleness of the tested samples during accelerated aging. The folding endurance measured after the modification has comparable values at both concentrations with the control (an unmodified sample). The stabilizing effect of tested systems is manifested only during accelerated aging. In the case of the control, after 15 days of accelerated aging, the number of double folds falls below a measurable level. Samples/paper easily “break” (they disintegrate) when they are handled. In the case of a lower concentration of DAO3 I., modification/stabilization caused a decrease in the number of double folds by up to 88% and a higher concentration of DAO3 II. by 56% during the accelerated aging (Table 1).

**Table 1:** Folding endurance for NOVO before modification (control) and after modification during accelerated aging.

Time	Folding Endurance [Double Folds ± sd]				
[Days]	Control	DAO3 I.	DAO3 II.	METE	DAO3 II. + METE
0	696 ± 221	568 ± 123	570 ± 160	382 ± 146	580 ± 100
3	148 ± 48	376 ± 105	220 ± 60	284 ± 82	300 ± 100
5	42 ± 17	180 ± 54	120 ± 58	327 ± 75	240 ± 70
10	5 ± 1	92 ± 36	140 ± 61	195 ± 52	210 ± 60
15	1 ± 0	67 ± 26	250 ± 80	144 ± 54	260 ± 130

sd—standard deviation.

The brittleness of modified NOVO samples immediately after modification, expressed by folding endurance (FE), was comparable for all three types of modifications (DAO3 II., METE, DAO3 II. + METE). During accelerated aging, there was a 55% decrease for both METE and DAO3 II. + METE modifications compared to the corresponding unmodified sample (Table 1).

Accelerated aging for the real BOOK samples caused a more significant decrease in FE values. The application of METE causes an 80% decrease in values compared to the modified unaged sample. The application MMS = DAO3 II. + METE causes an 89% decrease in values compared to the unaged modified sample (Table 2). The modification had the effect of reducing the brittleness of the tested paper during aging. We can talk about a more significant stabilization of acid lignin-containing paper.

**Table 2:** Folding endurance for BOOK before the modification (control) and after the modification during the accelerated aging.

Time	Folding Endurance [Double Folds ± sd]			
[Days]	Control	METE	DAO3 II.	DAO3 II. + METE
0	227 ± 105	155 ± 83	141 ± 62	207 ± 118
3	24 ± 12	72 ± 36	36 ± 22	112 ± 39
5	9 ± 5	61 ± 35	13 ± 5	101 ± 49
10	1 ± 1	42 ± 23	3 ± 2	33 ± 15
15	1 ± 0	31 ± 20	2 ± 1	22 ± 7

sd—standard deviation.

The effectiveness of the stabilization process can be evaluated as the coefficient of the relative increase of the lifetime for observed properties, in this case folding endurance (*S_τ,ω_*) [13,26,28]. It was determined as a linear regression of the logarithm of the folding endurance. The lifetime of the paper ends when the logarithm of the folding endurance becomes zero (*t_logω_*_=0_) for the reference sample (*n*) and the modified sample (*m*):

$$S_{\tau ,\omega }=\frac{t_{\log\omega =0,m}}{t_{\log\omega =0,n}}$$

The stabilization process under the terms of the Library of Congress and the Consortium Kniha^SK^ is considered effective if the coefficient of the relative increase of the lifetime reaches a value of at least 3 [10,23].

**Figure 2:**
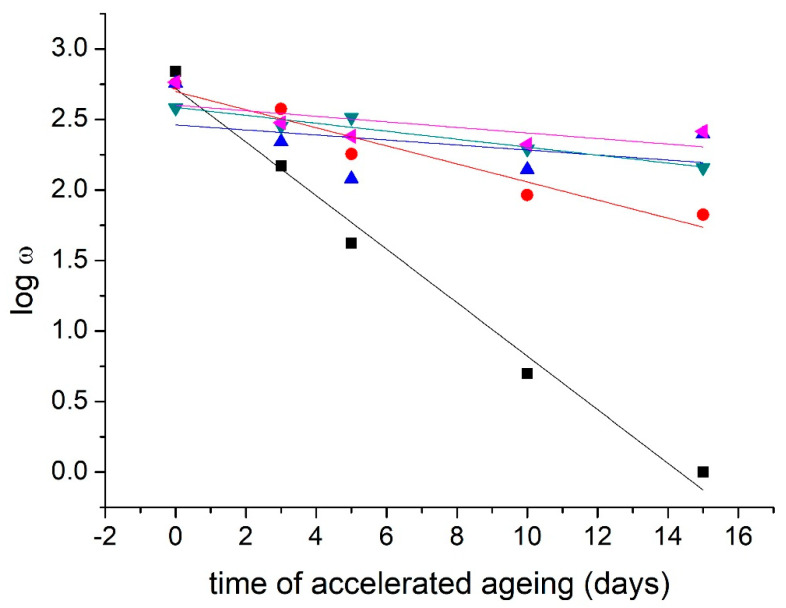
Stabilization effect of modification: Dependence of folding endurance from accelerated aging (t) for NOVO paper. ■—reference sample: logw = 2.719 − 0.189*t; R^2^ = 0.982; ●—DAO3 I.: logw = 2.698 − 0.064*t; R^2^ = 0.915; ▲—DAO3 II.: logw = 2.462 − 0.018*t; R^2^ = 0.122; ▼—METE: logw = 2.586 − 0.028*t; R^2^ = 0.917; ◄—DAO3 II. + METE: logw = 2.608 − 0.019*t; R^2^ = 0.281.

The values of the coefficient of the relative increase in the lifetime indicate the use of a higher concentration of the active substance/antioxidant (*S_τ,ω_* = 9.08) for the stabilization of traditional information carriers (Table 3, Figure 2).

**Figure 3:**
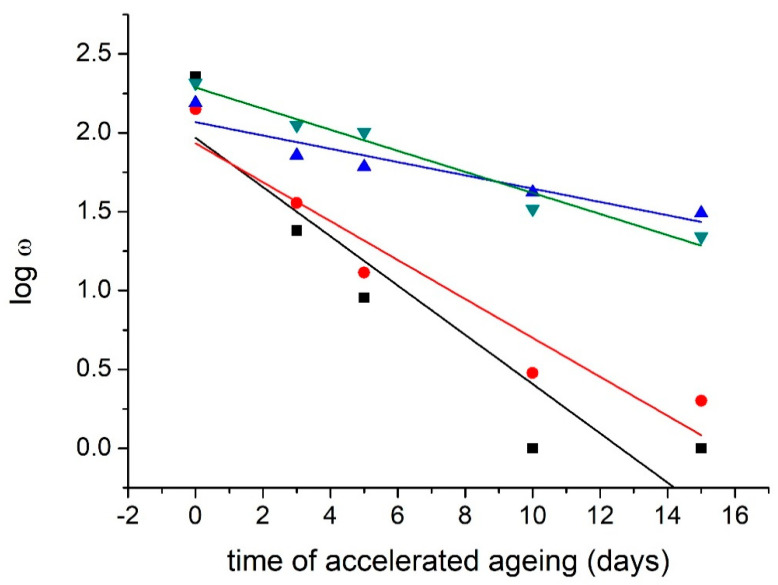
Stabilization effect of modification: Dependence of folding endurance on accelerated aging (t) for BOOK. ■—reference sample: logw = 1.955 − 0.148*t; R^2^ = 0.824; ●—DAO3 II.: logw = 1.954 − 0.127*t; R^2^ = 0.894; ▲—METE: logw = 2.066 − 0.042*t; R^2^ = 0.854; ▼—DAO3 II. + METE: logw = 2.282 − 0.066*t; R^2^ = 0.962.

The values of the coefficient of the relative increase in the lifetime show the suitability of using the DAO3 II. + METE modification in the case of NOVO paper (*S_τ,ω_* = 9.32) (Table 2, Figure 2). The theoretical lifetime of real aged paper (aging within the library collection + accelerated aging) was also extended by the effect of modification (Table 2, Figure 3).

In the case of using a modification system where the effect of an antioxidant and a deacidifying agent is combined, we observe a significant improvement in performance properties; fragility is reduced. Objectively, this shows the coefficient of the relative increase of the lifetime. The same trend was observed for the model test paper (NOVO) and the real book. An ANOVA was performed. Based on it, it is possible to conclude that the modifications of the NOVO paper (Table 1) are statistically significant (*p*-value = 1.84 × 10^−5^), but the aging time is not (*p*-value = 0.32). The real aged book shows statistically significant differences both in the case of modification (*p*-value = 7.04 × 10^−6^) and in the case of the aging time (*p*-value = 0.03).

### 3.2. Surface pH

Immediately after the modification/stabilization, the pH increases by 1 unit both in the case of DAO3 I. (pH = 5.60 ± 0,81) and DAO3 II. (pH = 5.42 ± 0.12) modification compared to the unmodified control sample (pH = 4.57 ± 0,12). After 15 days of accelerated aging, a decrease in pH was observed. At both antioxidant concentrations, the values stabilized at pH = 4.6 (DAO3 I. pH = 4.61 ± 0.29; DAO3 II. pH = 4.65 ± 0.21), whereas the control unmodified sample had a significantly more acidic pH = 3.19 ± 0.59.

Immediately after modification with a METE solution in HMDSO, the surface pH values increased to the expected alkaline range (pH = 8.89 ± 0.28) in the case of NOVO, as well as in the case of the real BOOK (pH = 8.71 ± 0.24). The increase was almost double in the case of NOVO (1.98×); in the case of BOOK samples, it was 1.8 times higher than the unmodified aged sample. In the alkaline range, the values were maintained even after accelerated aging—NOVO (pH = 7.4 ± 1.2) and BOOK (pH = 6.48 ± 0.31). The use of MMS (composed of the deacidifying and antioxidant component in a non-polar medium) increased the surface pH of the modified samples (NOVO pH = 8.98 ± 0.36; BOOK pH = 7.85 ± 0.39) compared to the control. Accelerated aging caused by MMS resulted in a 14% decrease in surface pH of both types of tested samples compared to the unaged NOVO and BOOK samples while in the case of METE (only deacidification), the decrease in pH was 17% (NOVO) and 25% (BOOK).

The shift in pH immediately after modification from a slightly alkaline to an alkaline range leads to the presumption of a sufficient alkaline reserve. Maintaining a slightly alkaline pH even after accelerated aging presupposes a slowing of the degradation of the main component of paper, cellulose, where degradation reactions occur at a significantly slower rate (i.e., in an alkaline environment) [29].

### 3.3. Gel Permeation Chromatography

The degradation of the main component of paper, cellulose, was observed on the break in the basis of the decay breaking the glycosidic bond that connects the glucopyranose units in the polymer chain, obtained from the Ekenstam equation [30]:

$$\frac{1}{{DP}_{t}}-\frac{1}{{DP}_{0}}=kt$$

**Figure 4:**
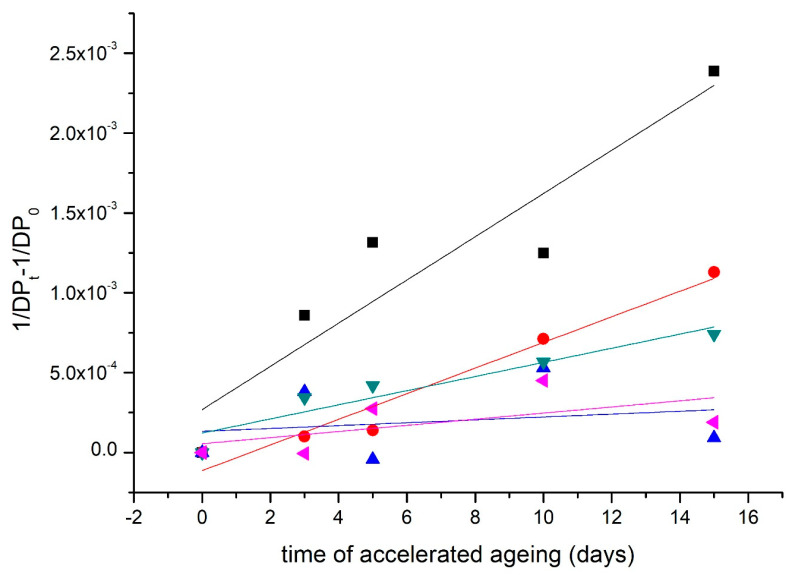
The rate of breaking of glycosidic bonds in cellulose during aging: Model lignin-containing acid test paper (NOVO). ■—reference sample; ●—DAO3 I.; ▲—DAO3 II.; ▼—METE; ◄—DAO3 II. + METE.

Modification with a higher concentration reduced the rate of cleavage of the glycosidic bond of cellulose by the order of 2 units compared to the control (Table 4, Figure 4).

**Table 4:** The rate of glycosidic bonds breaking in the cellulose.

Modification/Sample	NOVO [Day^−1^]	BOOK [Day^−1^]
Reference sample	1.35 × 10^−4^	5.74 × 10^−5^
METE	4.42 × 10^−5^	4.69 × 10^−6^
DAO3 I. (0.7% wt)	8.02 × 10^−5^	NM *
DAO3 II. (1% wt)	8.97 × 10^−6^	4.03 × 10^−5^
METE + DAO3 II.	1.92 × 10^−5^	2.49 × 10^−6^

* NM—not measured.

**Figure 5:**
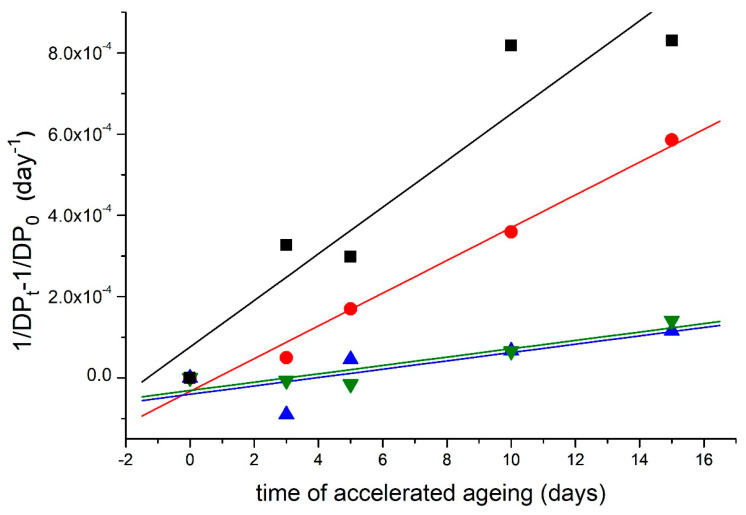
The rate of breaking of glycosidic bonds in cellulose during aging—samples of the BOOK. ■—reference sample; ●—DAO3 II.; ▲—METE, ▼—DAO3 II. + METE.

In the NOVO paper, the most significant decrease was observed in the modification with DAO3 II.; the decrease was 93%. For the BOOK, the most significant decrease in the rate of glycosidic bond breaking was observed in MMS composed with METE and DAO3 II.; the decrease was 96% (Figure 4 and Figure 5, Table 4).

Likewise, in this case, it is necessary to solve the stabilization of traditional media information comprehensively, not only by deacidification, but also by adding antioxidants.

### 3.4. Fourier Transform Infrared Spectroscopy/Oxidation Index

The oxidation index indicates the degree of oxidation of the material. It is the ratio of absorbance at wavenumbers 1730 cm^−1^ and 1620 cm^−1^ (A_1730 cm_^−1^/A_1620 cm_^−1^). Its increasing values indicate an increase in the oxidative degradation of the paper [31].

**Figure 6:**
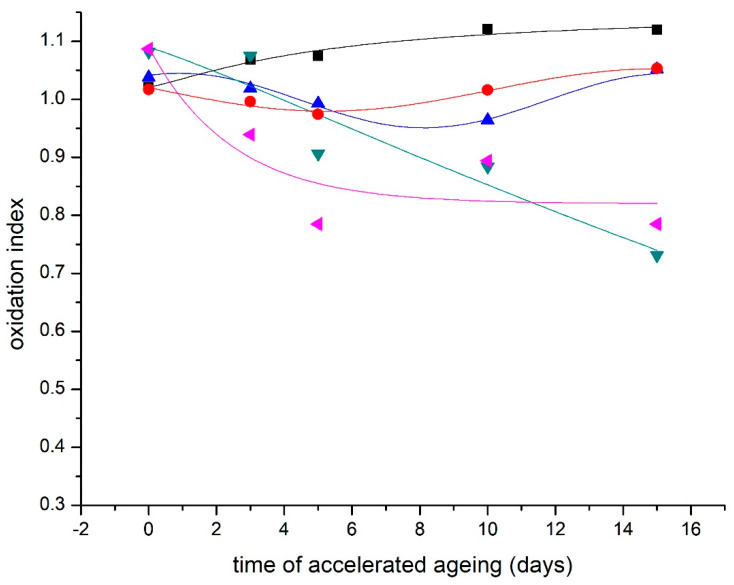
Changes in oxidation index of NOVO model paper during the accelerated aging. ■—reference sample; ●—DAO3 I.; ▲—DAO3 II.; ▼—METE; ◄—DAO3 II. + METE.

**Figure 7:**
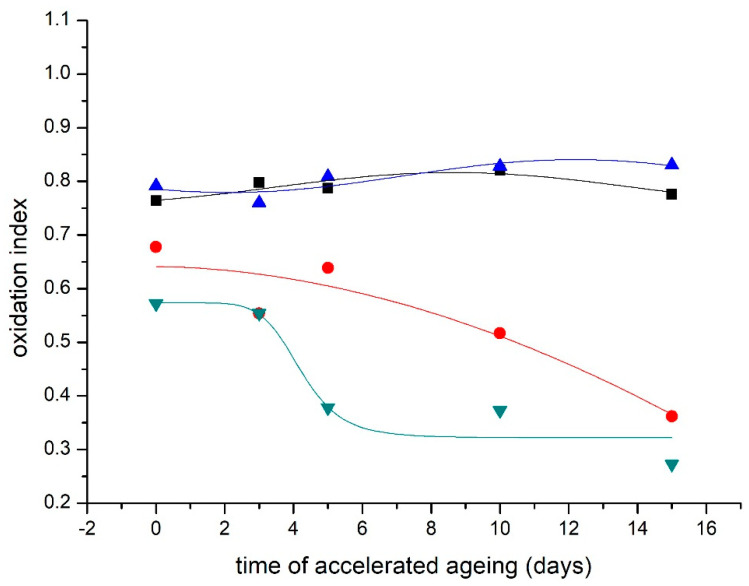
Changes in oxidation index of samples from the real BOOK during the accelerated aging. ■—reference sample; ●—DAO3 II.; ▲—METE, ▼—DAO3 II. + METE.

The modification causes a slight decrease in the oxidation index values compared to the unmodified control sample (Figure 6 and Figure 7).

## 4. Discussion

The main novelty of this study is the application of a new substance for increasing the mechanical, thermo-oxidative, and hydrolytic resistance of lignocellulosic information carriers (papers) made from 4-amino-2,2,6,6-tetramethylpiperidine or its mixture with a deacidifying agent consisting of an organometal or magnesium compound. This type of HALS was selected and synthesized to test a molecule that is an order of magnitude smaller than other known similar HALS, which react very willingly with alkoxy radicals and give non-radical products, and which would be able to penetrate the interior of the cell wall of pulp fibers down to the level of micro- and nanofibrils and fulfil the role of an antioxidant agent there. It is based on the reactions of the amino group with the carbonyl and carboxyl groups of partially thermo-oxidatively and photo-oxidatively degraded cellulose. A mutual condensation reaction produces imines or salts of carboxylic acids.

**Figure 8:**
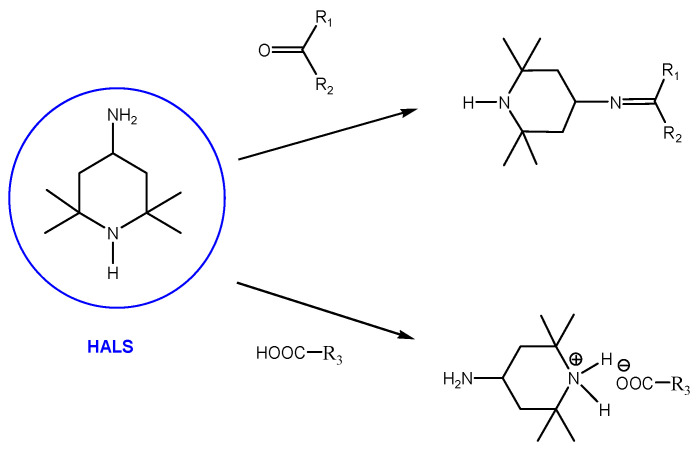
Scheme of the reaction of HALS with carbonyl and carboxyl compounds as products of thermo-oxidative and photo-oxidative degradation of cellulose macromolecule fragments (R1-, R2-, R3-).

HALS-type antioxidants can react with carbonyl and carboxyl compounds as products of thermo-oxidative and photo-oxidative degradation of cellulose macromolecule fragments (Figure 8). Carbonyl and carboxyl groups are formed by the oxidation of primary hydroxymethyl groups at position 6 and secondary hydroxyl groups at positions 2 and 3. A chemical fixation of the applied substance to the polymer can occur by covalent or ionic bonding to form an imine, which can further react with hydroxyl groups, especially -CH_2_ and -OH groups, to provide additional products. These may be the reason for increasing the mechanical strength of the polymer by some crosslinking [18,21,32,33].

**Figure 9:**
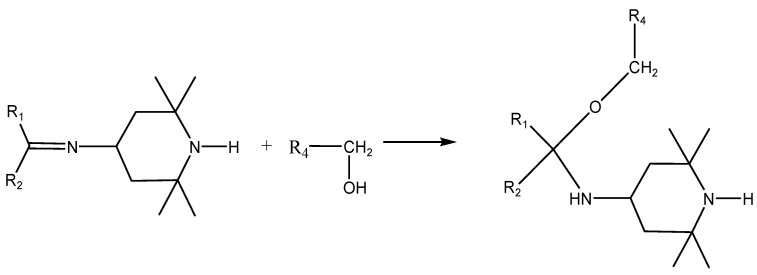
Scheme of the reaction of an imine with the hydroxyl groups of cellulose fragments (R1-, R2-, R4-) to form additional products.

In this way, the active substance can be chemically fixed to the cellulose by covalent or ionic bonds in the test paper. The resulting imine can further react with hydroxyl structures, especially -CH_2_ and -OH groups, to give additional products (Figure 9).

**Figure 10:**
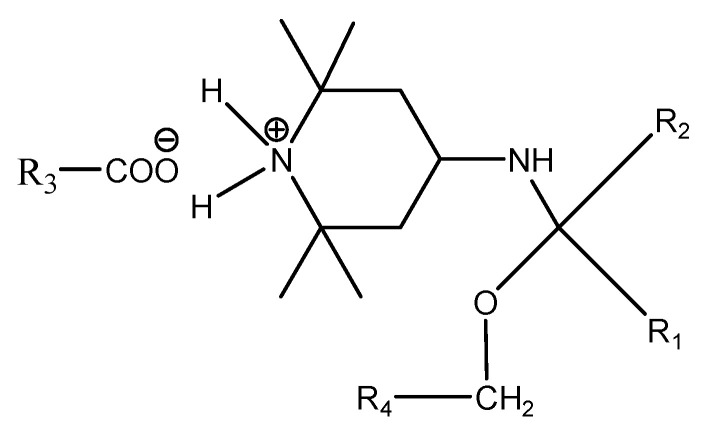
Possible cross-linking product of fragments of cellulose macromolecule (R1-, R2-, R3-, R4-) with 4-amino-2,2,6,6-tetramethylpiperidine.

This reaction (Figure 9) within the two cellulose fragments may be responsible for crosslinking and increasing the molecular weight, and it may be the explanation for the improvement of mechanical properties not only immediately after the application of the modifier, but also during accelerated aging. Of course, the combination of a covalently bound active agent on one part of the polymer chain and the ionic bond of the other cellulose chain fragment containing a carboxyl group can also contribute to this effect (Figure 10). The fragments of the cellulose macromolecule R1-R4 are, in principle, different but may ultimately be identical.

The achieved results point to the positive effect of the studied antioxidant on extending the life of acidic papers containing lignin. Oxidation index values indicate a decrease in oxidative degradation products and potential precursors of acid products, accelerating the statistical splitting of the cellulose chain. This is also related to the stabilization of DP and the change in the rate of cleavage of cellulose glycosidic bonds. The experimental measurements of the change in folding endurance support the proposed reaction schemes above (Figure 8, Figure 9 and Figure 10). Due to the antioxidants, paper brittleness is inhibited. This may be related to the cross-linking of cellulose chain fragments and, thereby, the stabilization of the pulp fiber structure.

## 5. Conclusions

The study was aimed at investigating the increase in the efficiency of the stabilization of acidic paper carriers of information by the application of a multifunctional modification system (MMS). An MMS contains components that eliminate not only the acids present and emerging in the paper, but also the products of oxidative degradation. The benefit is the use of the antioxidant 4-amino-2,2,6,6-tetramethylpiperidine series of hindered amines (HALS), which was selected and specified as suitable for creating a modifying system based on HMDSO. The main results are as follows:

- It was confirmed that the application of the system containing deacidification and an antioxidant component may increase the effectiveness of the protection of acid groundwood paper heritage objects;
- The addition of the tested antioxidant to the proposed deacidification system has a favorable effect on the stabilization of the pH value for a long amount of accelerated aging;
- The MMS provides inhibition of the oxidative degradation products’ formation and the stabilization of the cellulose polymerization degree;
- The importance of adding an antioxidant increases with the length of time the objects are aged; when growing, they need to eliminate oxidative degradation products;
- Modification does not significantly change the appearance of individual samples during the accelerated aging in comparison to the unmodified reference sample;
- Based on the research findings in the conservation of paper heritage objects, given the need to eliminate the causes (oxidation), consequences (acid hydrolysis), and expressions (brittleness) of material degradation, it is recommended to apply multifunctional modification systems in the field of preserving paper-based heritage objects.

## Figures and Tables

**Table 3:** Values of the coefficient of the relative increase of the lifetime for the folding endurance *S_τ,ω_* for NOVO and BOOK samples.

	NOVO		BOOK	
	*t_logw_*_=0_	*S_τ,ω_*	*t_logw_*_=0_	*S_τ,ω_*
reference	14.34		13.24	
DAO3 I.	42.12	2.94	NM *	NM *
DAO3 II.	130.28	9.08	49.00	3,70
METE	91.77	6.40	15.38	1.20
DAO3 II.+ METE	133.61	9.32	34.57	2.60

* NM—not measured.

## Data Availability

The data presented in this study are available on request from the corresponding author. The study contains summarized data from extensive experiments over 4 years. The amount of data, as well as their diversity, would not allow people not involved in this research to easily navigate within the dataset. The data are not publicly available due to high specificity of the research and since the authors are convinced that these data would not serve anyone else in research.
